# Overlapping Autoimmune Syndromes in Patients With Glial Fibrillary Acidic Protein Antibodies

**DOI:** 10.3389/fneur.2018.00251

**Published:** 2018-04-25

**Authors:** Xinguang Yang, Huiming Xu, Meilin Ding, Qingmei Huang, Baikeng Chen, Huacai Yang, Tianni Liu, Youming Long, Cong Gao

**Affiliations:** ^1^Department of Neurology, The Second Affiliated Hospital of Guangzhou Medical University, Guangzhou, China; ^2^Institute of Neuroscience, The Second Affiliated Hospital of Guangzhou Medical University, Key Laboratory of Neurogenetics and Channelopathies of Guangdong Province and the Ministry of Education of China, Collaborative Innovation Center for Neurogenetics and Channelopathies, Guangzhou, China; ^3^Department of Geriatrics, The First Affiliated Hospital of Sun Yat-sen University, Guangzhou, China

**Keywords:** glial fibrillary acidic protein, autoimmune, astrocytopathy, antibody, central nervous system

## Abstract

**Background:**

Glial fibrillary acidic protein (GFAP) astrocytopathy, an autoimmune central nervous system disorder with a specific GFAP-IgG, often coexists with other antibodies.

**Objective:**

The aim of this article was to study overlapping syndromes in autoimmune GFAP astrocytopathy.

**Methods:**

Antibody was detected by indirect immunofluorescence assay. Patient data were analyzed retrospectively.

**Results:**

Thirty patients with positive GFAP-IgG were included, of whom 10 were defined as overlapping syndrome. Four patients with positive aquaporin-4 (AQP4)-IgG, two with *N*-methyl-d-aspartate receptor-IgG, three with unknown neuronal antibodies, and one with double AQP4 and myelin oligodendrocyte glycoprotein-IgG were identified. GFAP-IgG and other specific antibodies occurred simultaneously at the initial attack in eight patients. The main symptoms included fever, headache, ataxia, psychosis, hypersomnia, dyskinesia, dementia, seizure, myelitis, and optical symptoms. Brain magnetic resonance imaging in four patients revealed characteristic radial enhancing patterns in the white matter. Cortical abnormalities were found in four patients. Other brain abnormalities occurred in the hypothalamus, midbrain, pons, medulla, cerebellum, and meninges. Six patients exhibited lesions in the spinal cord. In a subgroup study, patients with overlapping syndrome were younger at onset than those with non-overlapping syndrome.

**Conclusion:**

Overlapping antibodies are common in GFAP astrocytopathy.

## Introduction

Aquaporin-4 (AQP4) autoimmunity is associated with the central nervous system (CNS) astrocyte disorder neuromyelitis optica spectrum disorder (NMOSD) (1). Glial fibrillary acidic protein (GFAP) astrocytopathy is another severe immune-mediated inflammatory disorder of the CNS, which predominantly affects the meninges, brain, spinal cord, and optic nerve. The disease is associated with antibodies against GFAP, and the detection of GFAP-IgG is helpful for diagnosis of GFAP astrocytopathy compared with other inflammatory autoimmune disorders of the CNS (2, 3).

Overlapping autoimmune syndromes occur commonly in patients with antibody-mediated inflammatory disorders. NMOSD with positive AQP4 antibody often has a concurrent autoimmune disease, such as Sjögren’s syndrome, myasthenia gravis, or systemic lupus erythematosus (4, 5). A few studies have reported that anti-*N*-methyl-d-aspartate receptor (NMDAR) encephalitis is associated with acute demyelinating encephalomyelitis, myelitis, or NMOSD (6). Recently, a Mayo clinic study (3) found that GFAP-IgG was accompanied by coexisting NMDA-R-IgG, AQP4-IgG, or both. Therefore, coexistence of autoantibodies in GFAP astrocytopathy is not rare.

However, GFAP astrocytopathy is poorly understood, and only limited reports have described the overlapping syndromes. In our previous studies (7, 8), we described a group of patients with GFAP astrocytopathy. In this study, we have recruited additional patients with GFAP astrocytopathy, of whom 10 had overlapping syndromes, and focused on the clinical data, magnetic resonance imaging (MRI) features, and cerebral spinal fluid (CSF) examination.

## Patients and Methods

### Patients

This retrospective study was approved by the Ethics Committee of the Second Affiliated Hospital of Guangzhou Medical University, China. Data analysis was performed based on the Chinese laws for data protection. All patients provided written informed consent.

From June 2013 to September 2017, paired samples of serum and CSF were prepared for study as described in the previous study (8). For this study, serum and CSF samples from 749 Chinese patients with inflammatory and non-inflammatory symptoms were collected and routinely tested for autoantibodies. All samples were collected during the early active disease stage, prior to treatment. No healthy controls were included in our study, because of a lack of CSF samples for these individuals.

We detected positive GFAP antibody in 30 patients. None of these patients had a history of neoplasm. The 28 control patients (female/male, 27/1; median age of onset, 41 years) with positive AQP4 antibody were diagnosed as NMOSD according to published criteria (9) and were negative for GFAP antibody. Data acquired from the first admission record included age, sex, medication, MRI features, and clinical characteristics.

### Anti-GFAP Antibody Detection in CSF and Serum

Tissue-based and cell-based assays were used, as described in our previous study (8). Antibody detection was performed using an indirect immunofluorescence assay on rat hippocampal and cerebellar tissue. Samples were retested in a cell-based assay using HEK293 cells transfected with GFAPα and GFAPε genes.

### Testing of Other Antibodies

The presence of AQP4-M1, AQP4-M23, myelin oligodendrocyte glycoprotein (MOG), collapsin response mediator protein 5, Hu, Yo, Ri, amphiphysin, Ma2, NMDAR, α-amino-3-hydroxy-5-methyl-4-isoxazolepropionic acid (AMPA) receptor 1, AMPA2, leucine-rich glioma-inactivated 1, γ-aminobutyric acid type-A receptor, and glutamic acid decarboxylase 65 antibodies was assessed in patients with GFAP antibodies. Detection was carried out as described in previous studies (7, 8).

### Statistical Analysis

All statistical analyses were performed using the Statistical Program for Social Sciences software (version 17.0; SPSS, Inc., Chicago, IL, USA). Statistical analyses were performed with the Fisher’s exact test for binary and categorical data. The Mann–Whitney test was used for continuous variables, and *p* < 0.05 was considered statistically significant.

## Results

### Patient Demographics and Clinical Data

A total of 30 patients positive for GFAP antibodies and with detailed data were included in this retrospective study. This cohort comprised 21 females and 9 males (a female to male ratio of 2.33). Their median age at onset was 46 years (range, 19–77 years), of whom eight patients (26.7%) were older than 60 years at disease onset.

**Figure 1:**
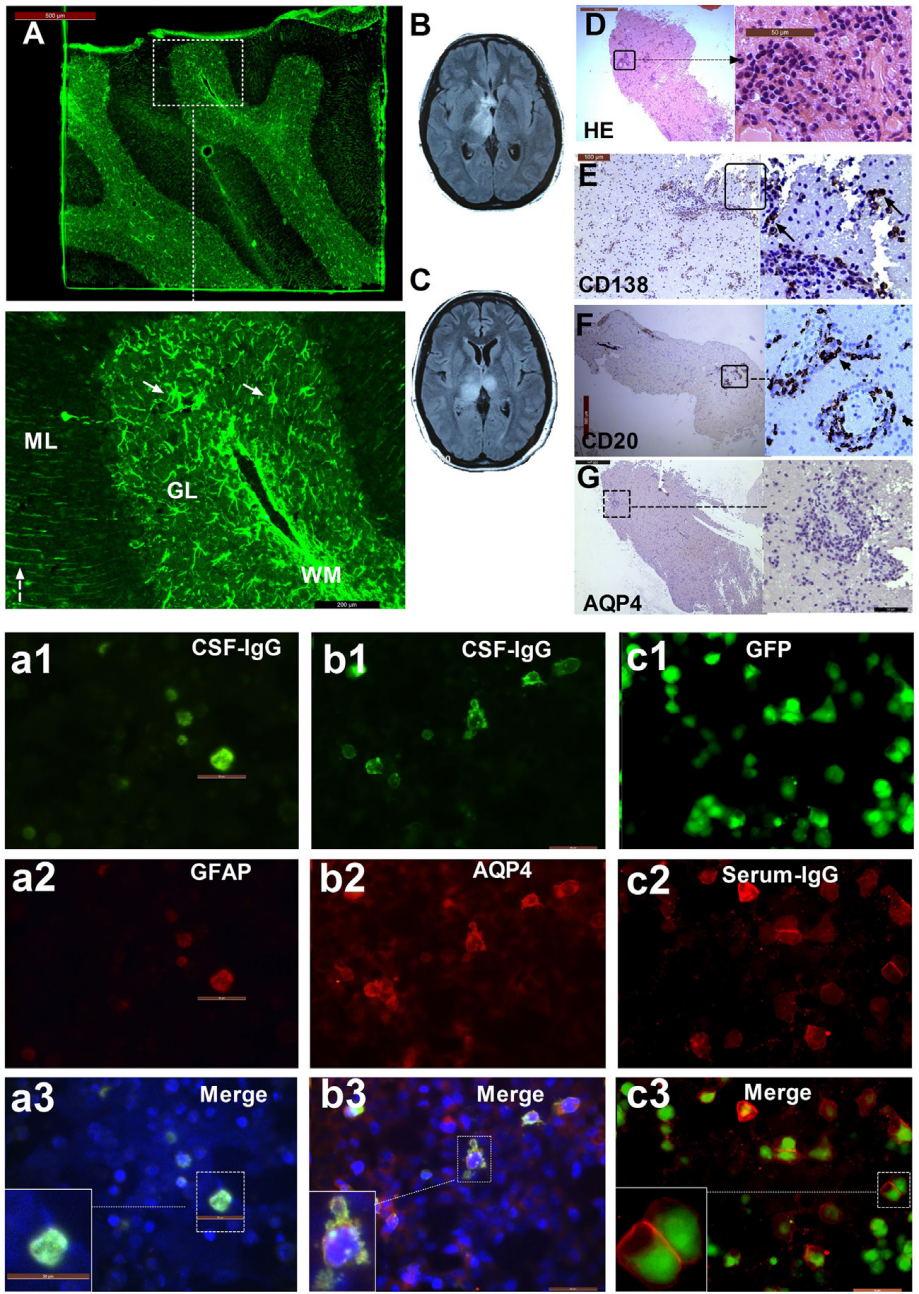
The patient with three types of antibodies: immunofluorescence pattern, magnetic resonance imaging (MRI), and neuropathological features. **(A)** Cerebellum section showing strong immunofluorescence in astrocytes (arrow) in white matter (WM), the granular layer (GL), and the molecular layer with a Bergmann radial pattern (dashed arrow); **(a1)** CSF-IgG bound to HEK293 cells transfected with GFAPε, showing the microfilament pattern (green); **(a2)** glial fibrillary acidic protein (GFAP) α (red) expression in HEK293 cells transfected with GFAPα, showing the microfilament pattern; **(a3)** merged image from **(a1)** and **(a2)**. **(b1)** CSF-IgG bound to HEK293 cells transfected with AQP4 (green); **(b2)** AQP4-M23 protein (red) expression in HEK293 cells transfected with AQP4; **(b3)** merged image from **(b1)** and **(b2)**. **(c1)** HEK293 cells stably expressing green fluorescent protein (GFP)-tagged myelin oligodendrocyte glycoprotein (MOG) isoforms (green); **(c2)** transfected cells immunostained with human IgG (red); **(c3)** merged image from **(c1)** and **(c2)**. **(B)** An MRI scanning shows lesions in the left thalamus and anterior commissure at the first attack. **(C)** Bilateral thalamus lesions at the third attack. **(D)** Hematoxylin-eosin staining of brain biopsy tissue shows extensive infiltration of inflammatory cells, especially around the vessels. **(E)** Extensive anti-CD138-positive cells. **(F)** Strong immunostaining for anti-CD20 is found in lesions around blood vessels. **(G)** Almost all AQP4 staining is lost.

**Figure 2:**
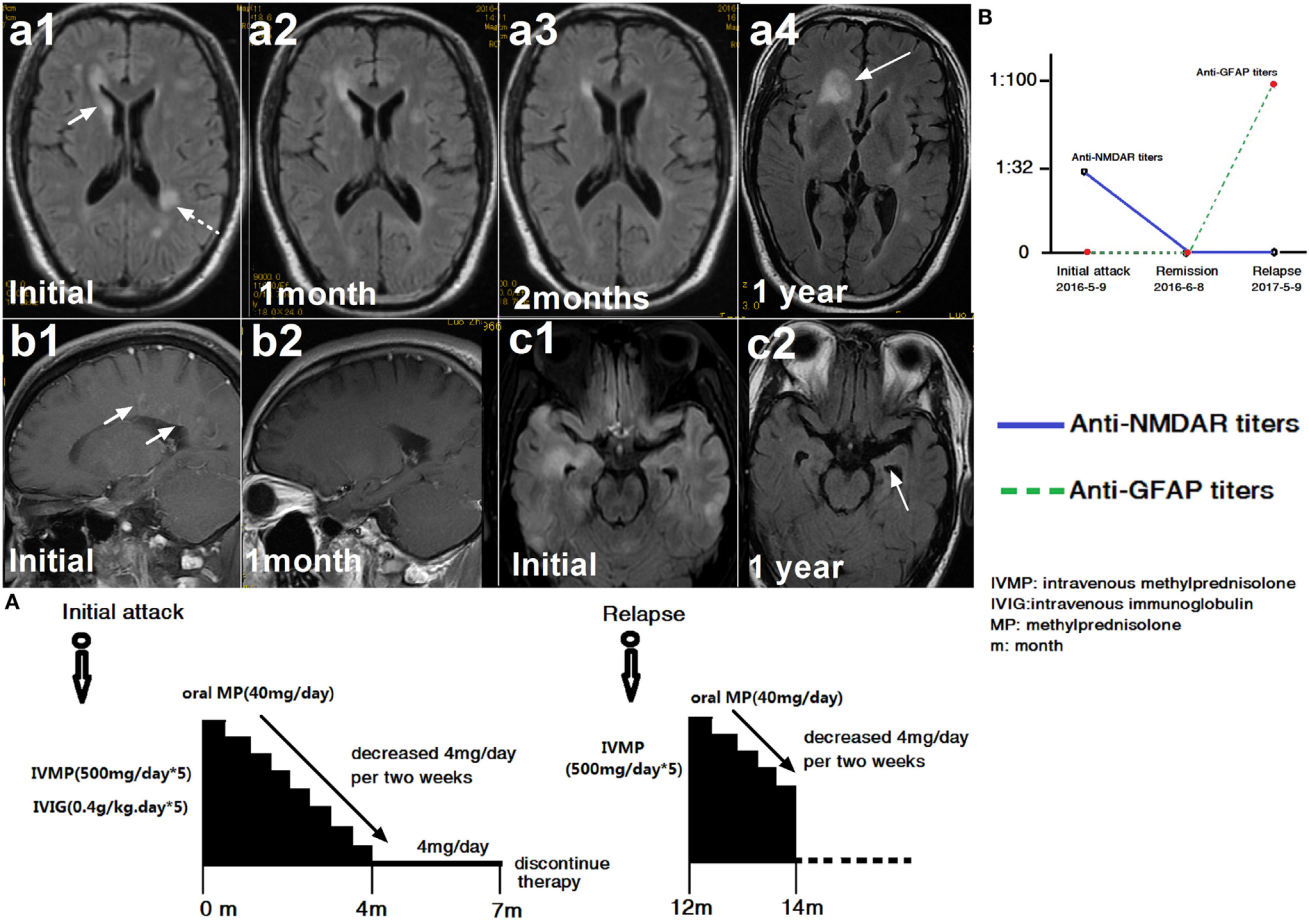
A patient with glial fibrillary acidic protein (GFAP) astrocytopathy following anti-*N*-methyl-d-aspartate receptor (NMDAR) encephalitis. **(a1–c2)** Brain magnetic resonance imagings (MRIs). **(A)** Treatment course. **(B)** NMDAR and GFAP antibody titers. The first brain MRI showed lesions in the right caudate nucleus, white matter along the ventricle **(a1)** and enhancement around vessels **(b1)**. At this time, cerebral spinal fluid (CSF) showed positive NMDAR-IgG at 1:32 titer. The patient was treated with intravenous methylprednisolone (500 mg/day for 5 days) and intravenous immunoglobulin (0.4 g/kg for 5 days). At follow-up, an MRI scanning showed improvement in the brain abnormalities **(a2, a3)** and the disappearance of enhancement **(b2)**. One month later, NMDAR-IgG and GFAP-IgG were negative. However, the patient relapsed 1 year later due to discontinuation of oral steroid. At this time, the MRI showed a large lesion in the frontal lobe **(a4)**. Compared with the initial MRI scan, left hippocampal atrophy **(c1, c2)** was found. At this time, CSF was positive for GFAP antibody but negative for NMDAR antibody **(B)**.

Among these 30 participants, 10 patients (33.3%) with other specific antibodies were described as overlapping syndrome in this study (Table 1). Of them, four patients were positive for AQP4 antibody, two were positive for NMDAR antibody, three patients had an unknown neuronal antibody, and one patient was positive for both AQP4 and MOG antibodies (Table 1). The patients who had three different kinds of antibody (GFAP, AQP4, and MOG) underwent brain pathological examination (Figure 1). The pathological examination showed extensive inflammatory cells, including CD20^+^, CD3^+^, and CD138^+^ cells, around the vessels. Immunohistochemical analysis also showed complete loss AQP4 and GFAP. However, MOG expression was preserved, and no obvious demyelination could be found in this patient. Anti-GFAP antibody and other specific antibodies that occurred simultaneously at the initial attack were found in eight patients. One patient developed GFAP astrocytopathy following NMO (10 years later), and another patient developed GFAP astrocytopathy following NMDAR encephalitis (1 year later; Figure 2).

**Table 1:** Data of 10 patients with autoimmune overlapping syndromes.

No	Sex	Age (years)	Duration	Relapse	Overlapping antibodies occur simultaneously	Symptoms	Other serum antibody	CSF abnormality	MRI features
1	F	41	1.5 years	Yes	Yes	Fever, SIADH, vertigo, quadriplegia, dementia, psychosis	ANA, SSA, Ro52, pANCA,^a^ MOG	WBC: 20 cells/mm^3^; protein: 1.007 g/L; GFAPε-IgG; AQP4-IgG	Brain: Bilateral thalamus, right hypothalamus and midbrain, left hippocampus, right parietal, frontal lobe, and cerebellum T1-weighted “radial enhancing” Sc: normality
2	M	57	1.5 years	No	Yes	Dementia, ON, LETM, hypersomnia	ANA, pANCA,^a^ SSA, Ro52	WBC: 145 cells/mm^3^; protein: 1.781 g/L; GFAPα + ε-IgG; neuronal antibody(+)	Brain: Diffuse lesion in white mater, midbrain, and basal ganglion, pon, hippocampus and cerebellum Sc: C1-T12
3	F	27	1 years	No	Yes	Fever, headache, seizures, ON, LETM, ataxia, psychosis	ANA, pANCA^a^	WBC: 247 cells/mm^3^; protein: 1.982 g/L; GFAPα + ε-IgG; neuronal antibody(+)	Brain: Lesions in left temporal lobe, meningeal abnormality Sc: C1-T12 Meningeal abnormality
4	F	35	1 year	No	Yes	LETM	ANA, AQP4, GFAPα + ε-IgG,	WBC: 65 cells/mm^3^; protein: 0.625 g/L; AQP4-IgG	Brain: Normality Sc: T5-T11
5	F	27	10 years	Yes	Followed NMO	ON, LETM, hypersomnia	ANA, AQP4	WBC: 24 cells/mm^3^; protein: 0.65 g/L; AQP4-IgG; GFAPα + ε-IgG	Brain: Bilateral thalamus, bilateral hypothalamus, midbrain, pons and medulla Sc: C1-C3
6	M	40	1 year	No	Followed NMDAR encephalitis	Headache, dementia, psychosis, dyssomnia	ANA, SSB, AMA-M2 RO52	WBC: 10 cells/mm^3^; protein: 0.866 g/L; NMDAR-IgG; GFAPα + ε-IgG	Brain: Right caudate nucleus, right frontal lobe, white matter abnormality around the lateral ventricle, bilateral hippocampus, and cortex abnormality. T1-weighted “radial enhancing” Sc: Normality
7	M	19	1 year	No	Yes	Fever, headache, psychosis, ON	AQP4-IgG, GFAPα + ε-IgG,	WBC: 33 cells/mm^3^; protein: 0.513 g/L; GFAPα + ε-IgG; AQP4-IgG	Brain: Right hippocampus, thalamus, hypothalamus, posterior limb of internal capsule, and midbrain T1-weighted “radial enhancing” Sc: NA Meningeal abnormality
8	M	43	1 year	Yes	Yes	Fever, headache, psychosis, hypersomnia, epilepsy, dyskinesia	Negative	WBC: 38 cells/mm^3^; protein: 0.501 g/L; GFAPα + ε-IgG; NMDAR-IgG	Brain: Bilateral hippocampus, right white matter around the ventricle T1-weighted “radial enhancing” Sc: Normality
9	F	19	1 year	Yes	Yes	ON, LETM, Dementia	RO52, SSA AMA-M2, AQP4-IgG, GFAPα + ε-IgG,	WBC: 30 cells/mm^3^; protein: 0.421 g/L; AQP4-IgG; GFAPα + ε-IgG	Brain: Non-specific lesions in white matter Sc: C1–T7
10	F	23	3 years	Yes	Yes	Fever, headache, epilepsy, LETM, ON	Negative	WBC: 8 cells/mm^3^; protein: 0.147 g/L; GFAPα + ε-IgG; neuronal antibody	Brain: Bilateral hippocampus, left thalamus, hypothalamus, right lesion in white matter lesion around the forth ventricle posterior limb of internal capsule Sc: C1–C6, T4–T8

*ACLA, anticardiolipin antibody; AECA, anti-endothelial cell antibody; ANA, antinuclear antibody; AQP4, aquaporin-4; cANCA, cytoplasmic antineutrophil cytoplasmic antibody; CSF, cerebral spinal fluid; Ds-DNA, double-stranded DNA; F, female; LETM, longitudinally extensive transverse myelitis; M, male; MOG, myelin oligodendrocyte glycoprotein; NA, no application; NMDAR, N-methyl-d-aspartate receptor; ON, optic neuritis; GFAP, glial fibrillary acidic protein; pANCA, perinuclear antineutrophil cytoplasmic antibodies; RO52, Ro-52 antibodies; Sc, spinal cord; SIADH, syndrome of inappropriate secretion of antidiuretic hormone; SSA, Sjögren’s syndrome A; WBC, white blood cell*.

*^a^All the ANCA were negative for MPO and PR3 antigen*.

The patients with overlapping syndrome included six females and four males, whose median age at onset was 31 years (19–57 years). The main brain symptoms observed included fever (*n* = 5), headache (*n* = 5), ataxia (*n* = 1), psychosis (*n* = 5), hypersomnia (*n* = 3), dyskinesia (*n* = 1), dementia (*n* = 4), seizure (*n* = 3), myelitis (*n* = 6), and optical symptoms (*n* = 6). CSF abnormalities were found in nine patients. They all received brain MRI scans, and eight of these patients showed brain abnormalities (80%). Brain MRI in four patients (40%) revealed characteristic radial enhancing patterns in the white matter around the ventricle. Cortical abnormalities were found in four patients (40%) by MRI. Other brain abnormalities occurred in the hypothalamus (4, 40%), midbrain (4, 40%), pons (2, 20%), medulla (2, 20%), cerebellum (2, 20%), and meninges (2, 20%). Six of these patients (60%) exhibited lesions in the spinal cord, of whom two patients had very long lesions from the cervical segment to lumbar segment of spinal cord.

### Comparison of GFAP Astrocytopathy and AQP4 Astrocytopathy Phenotypes at Onset

According to their overlapping status, 30 patients with positive GFAP antibody were divided into 2 subgroups: overlapping syndrome (*n* = 10) and non-overlapping syndrome (*n* = 20). In the GFAP astrocytopathy subgroup study, patients with overlapping syndrome were younger at onset than those with non-overlapping syndrome (median age, 52 vs 31 years, *p* = 0.0046). However, no further differences could be confirmed between the two subgroups.

To compare the difference between GFAP astrocytopathy and AQP4 astrocytopathy, 28 patients with positive AQP4 antibody who had initial data and were negative for GFAP antibody were recruited as controls. The GFAP astrocytopathy group had fewer females than the AQP4 astrocytopathy group (female/male, 21/9 vs 27/1, *p* = 0.012), more fever (56.7 vs 3.6%, *p* = 0.00003), headache (70 vs 0%, *p* = 0.0000000), psychosis (33.3 vs 0%, *p* = 0.0008), neuronal antibody (20 vs 0%, *p* = 0.002), higher CSF white blood cell counts (median count 33 vs 7 cells/mm^3^, *p* = 0.0017), higher protein levels (0.79 *vs* 0.43 g/L, *p* = 0.021), more “radial pattern” abnormalities with enhancement (46.7 vs 3.7%, *p* = 0.0002), cortex abnormalities (30 vs 3.7%, *p* = 0.0125), meningeal abnormalities (20 vs 0%, *p* = 0.024), and whole spine abnormalities (27.2 vs 0%, *p* = 0.001). The comparison of GFAP subgroups and AQP4 astrocytopathy is shown in Table 2.

**Table 2:** A comparison between patients with GFAP and AQP4 astrocytopathy at onset.

	All GFAP astrocytopathy	Non-overlapping syndrome	Overlapping syndrome	AQP4 astrocytopathy	P1	P2	P3	P4
*n*	30	20	10	28	–	–	–	–
Female/male	21/9	15/5	6/4	27/1	NS	0.012	NS	0.012
Median age at onset, years (range)^a^	46 (19–77)	52 (24–77)	31 (19–57)	41 (16–72)	0.005	NS	<0.0001	NS
Fever, *n* (%)	17 (56.7)	12 (60)	5 (50)	1 (3.6)	NS	<0.0001	<0.0001	0.003
Brain symptoms, *n* (%)	27 (90)	18 (90)	9 (90)	7 (25)	NS	<0.0001	<0.0001	0.0005
Headache, *n* (%)	21 (70)	16 (80)	5 (50)	0	NS	<0.0001	<0.0001	0.0005
Ataxia, *n* (%)	5 (16.7)	4 (20)	1 (10)	4 (14.3)	NS	NS	NS	NS
Psychosis, *n* (%)	10 (33.3)	5 (25)	5 (50)	0	NS	0.0008	0.009	0.0005
Hypersomnia, *n* (%)	4 (13.3)	1 (5)	3 (30)	3 (10.7)	NS	NS	NS	NS
Dyskinesia, *n* (%)	3 (10)	2 (10)	1 (10)	0	NS	NS	NS	NS
Dementia, *n* (%)	6 (20)	2 (10)	4 (40)	0	NS	0.024	NS	0.003
Seizure, *n* (%)	6 (20)	3 (15)	3 (30)	0	NS	0.024	NS	0.014
Myelitis, *n* (%)	19 (63.3)	13 (75)	6 (60)	25 (89.3)	NS	0.031	NS	NS
Optic symptoms, *n* (%)	18 (60)	12 (60)	6 (60)	23 (82.1)	NS	NS	NS	NS
Neuronal antibody, *n* (%)	6 (20)	0	6 (60)	0	0.0004	0.024	–	<0.0001

CSF examination								
WBC count (cells/mm^3^)	33 (5–305)	37 (5–305)	32 (8–247)	7 (0–98)	NS	0.002	0.012	0.032
Protein level (g/L)	0.79 (0.147–2.99)	0.81 (0.21–2.99)	0.64 (0.147–1.982)	0.43 (0.135–1.4)	NS	0.021	0.01	NS
MRI features, *n* (%)								
Brain abnormality	24 (80)	16 (80)	8 (80)	18 (64.3)	NS	NS	NS	NS
Radial enhancement	14 (46.7)	10 (50)	4 (40)	1 (3.7)	NS	0.0002	0.0002	0.012
Cortex	9 (30)	5 (25)	4 (40)	1 (3.7)	NS	0.012	NS	0.012
Hypothalamus	7 (23.3)	3 (15)	4 (40)	7 (25)	NS	NS	NS	NS
Midbrain	11 (36.7)	7 (35)	4 (40)	6 (21.4)	NS	NS	NS	NS
Pons	13 (43.3)	11 (55)	2 (20)	6 (21.4)	NS	NS	0.03	NS
Medulla	8 (26.7)	6 (30)	2 (20)	10 (35.7)	NS	NS	NS	NS
Cerebellum	8 (26.7)	6 (30)	2 (20)	3 (10.7)	NS	NS	NS	NS
Meningeal abnormality	6 (20)	4 (20)	2 (20)	0	NS	0.024	0.025	0.064

Spinal cord abnormality (%)								
Cervical lesion	15; 15/27 (55.6)	12; 12/17 (70.6)	3; 3/10 (30)	20; 20/28 (71.4)	0.057	NS	NS	0.030
Thoracic lesion	11; 11/27 (40.7)	8; 8/17 (47.1)	3; 3/10 (30)	9; 9/28 (32.1)	NS	NS	NS	NS
Whole spinal abnormality	6; 6/27 (22.2)	4; 4/17 (23.5)	2; 2/10 (20)	0	NS	0.001	0.016	0.064

*NS, no significance; P1, overlapping syndrome compared with non-overlapping syndrome; P2, GFAP astrocytopathy compared with AQP4 astrocytopathy; P3, overlapping syndrome compared with AQP4 astrocytopathy; P4, non-overlapping syndrome compared with AQP4 astrocytopathy*.

## Discussion

In previous Mayo clinic reports (2, 3), Lennon and her colleagues identified patients with GFAP astrocytopathy with several additional kinds of autoantibody, including NMDAR antibody, AQP4 antibody, and MOG antibody, which suggests an immune encephalitis or demyelinating disorder. They found that 41 patients had one or more coexisting antibodies in serum or CSF (40%), of which NMDAR-IgG was the most common coexisting antibody, and AQP4-IgG was the next most common. In this study, we found that 10 of 30 patients (33.3%) had a coexisting antibody, supporting the finding that coexisting antibodies are common in patients with GFAP astrocytopathy. In addition to the two patients with NMDAR antibody, it is interesting that we also found three patients with an unknown neuronal antibody. Other antibodies such as AQP4 or MOG, in association with GFAP antibodies, were found in another five patients, which was similar to the above study (3).

Although both GFAP and AQP4 astrocytopathy have specific IgGs targeting astrocytes and are involved in myelitis, optic neuritis, and brain symptoms, it seems that GFAP astrocytopathy is quite different from AQP4 astrocytopathy in many clinical manifestations, including fever, headache, and psychiatric symptoms, suggesting immune encephalitis features (2, 3). Furthermore, our results also showed that obvious findings were different in initial MRI patterns between GFAP and AQP4 astrocytopathy. In patients with GFAP astrocytopathy, almost half of the patients with lesions had the radial enhancement pattern. By contrast, only one patient with AQP4 astrocytopathy had such enhancement. A striking pattern of linear perivascular radial gadolinium enhancement in 53% of patients was described in a recent study (3), similar to our present results. In the two subgroups of GFAP astrocytopathy, similar differences could be seen between the subgroups and AQP4 astrocytopathy. Therefore, our data indicate that different immune mechanisms were shared in GFAP and AQP4 astrocytopathy. However, although two or more immune mechanisms may occur in GFAP astrocytopathy with overlapping syndrome, no further differences could be identified between the patients with and without overlapping syndrome, except for the age at onset. Therefore, the exact difference between the two kinds of patients with GFAP astrocytopathy is unknown. However, we only had 10 patients with overlapping syndrome, which represents a very small sample size, and future studies should be undertaken in a larger population.

This study provides some interesting findings and issues that are important for clinical diagnosis and recognition. In our 10 patients with overlapping syndrome, 2 cases developed GFAP astrocytopathy separated in time from the episode of anti-NMDAR encephalitis or AQP4 astrocytopathy, making it easy to recognize and draw a definite diagnosis. However, there were eight patients with GFAP antibodies occurring simultaneously with clinical and MRI features of autoimmune encephalitis or demyelinating disorders. This could be a confounding condition for clinicians at the initial episode. Based on the clinical manifestations and positive AQP4-IgG, five patients could be diagnosed as NMOSD. The patient with three kinds of antibodies who underwent pathological examination and showed typical extensive AQP4 loss also met the pathological criterion of positive anti-AQP4 NMOSD. The other three patients could be diagnosed as autoimmune encephalitis, according to the diagnostic criteria (10) and positive neuronal antibody. These mixed phenotypes suggest the coexistence of two simultaneously active immune mechanisms, which has been described in NMDAR encephalitis with AQP4 or MOG antibodies (5). Therefore, as overlapping antibodies occur simultaneously at onset in patients with autoimmune encephalitis or demyelinating disorders, suitable diagnosis and classification is a clinical challenge. Diagnostic criteria for GFAP astrocytopathy should be designed in the future.

In conclusion, we found that 10 of 30 patients with GFAP-IgG harbor additional antibodies. Therefore, overlapping autoantibodies are common in GFAP astrocytopathy, involving AQP4-IgG, MOG-IgG, NMDAR-IgG, or other neuronal antibodies. In this study with small sample numbers, our results suggest that there is no critical difference between patients with and without overlapping syndrome.

## Ethics Statement

This retrospective study was approved by the Ethics Committee of the Second Affiliated Hospital of Guangzhou Medical University, China. Data analysis was performed based on the Chinese laws for data protection.

## Author Contributions

All authors listed have made a substantial, direct and intellectual contribution to the work, and approved it for publication.

## Conflict of Interest Statement

The authors have declared that no competing interests exist. The reviewer A-KP and handling Editor declared their shared affiliation.
